# CD39^+ ^Regulatory T cells suppress generation and differentiation of Th17 cells in human malignant pleural effusion via a LAP-dependent mechanism

**DOI:** 10.1186/1465-9921-12-77

**Published:** 2011-06-10

**Authors:** Zhi-Jian Ye, Qiong Zhou, Jian-Chu Zhang, Xiao Li, Cong Wu, Shou-Ming Qin, Jian-Bao Xin, Huan-Zhong Shi

**Affiliations:** 1 Department of Respiratory Diseases, Key Laboratory of Pulmonary Diseases of Health Ministry, Union Hospital, Tongji Medical College, Huazhong University of Science and Technology, China; 2 Institute of Respiratory Diseases, First Affiliated Hospital, Guangxi Medical University, China

**Keywords:** latency-associated peptide, malignant pleural effusion, regulatory T cells, Th17 cells, transfer growth factor

## Abstract

**Background:**

Both regulatory T cells (Tregs) and T helper IL-17-producing cells (Th17 cells) have been found to be involved in human malignancies, however, the possible implication of Tregs in regulating generation and differentiation of Th17 cells in malignant pleural effusion remains to be elucidated.

**Methods:**

The numbers of both CD39^+^Tregs and Th17 cells in malignant pleural effusion and peripheral blood from patients with lung cancer were determined by flow cytometry. The regulation and mechanism of Tregs on generation and differentiation of Th17 cells were explored.

**Results:**

Both CD39^+^Tregs and Th17 cells were increased in malignant pleural effusion when compared with blood, and the numbers of CD39^+^Tregs were correlated negatively with those of Th17 cells. It was also noted that high levels of IL-1β, IL-6, and TGF-β1 could be observed in malignant pleural effusion when compared the corresponding serum, and that pleural CD39^+^Tregs could express latency-associated peptide on their surface. When naïve CD4^+ ^T cells were cocultured with CD39^+^Tregs, Th17 cell numbers decreased as CD39^+^Treg numbers increased, addition of the anti-latency-associated peptide mAb to the coculture reverted the inhibitory effect exerted by CD39^+^Tregs.

**Conclusions:**

Therefore, the above results indicate that CD39^+^Tregs inhibit generation and differentiation of Th17 cells via a latency-associated peptide-dependent mechanism.

## Introduction

It has been well documented that CD4^+ ^T lymphocyte dominance occurs in malignant pleural effusion (MPE) [1,2]. On encountering an antigen, naïve CD4^+ ^T-helper precursor cells enact a specific process that results in differentiation toward the T-helper type 1 (Th1) or Th2 lineage. Early studies have suggested that Th1/Th2 cell balance in MPE may influence pathophysiologic process of pleural disease [3,4]. Two additional CD4^+ ^T cell subsets, regulatory T cells (Tregs) and T helper IL-17-producing cells (Th17 cells), have been described more recently. Tregs are characterized by the expression of the lineage-specific transcription factor FOXP3, which is involved both in their development and in their suppressor functions [5,6]. Th17 cells are now defined as a separate subset distinct from the Th1, Th2, and Tregs, in terms of developmental regulation and function [7,8]. Our previous studies showed that increased Tregs were found in MPE, and these Tregs were recruited into pleural space induced by chemokine CCL22 [9,10]. More recently, we have demonstrated that due to local differentiation and expansion stimulated by cytokines and to recruitment from peripheral blood induced by chemokines, the numbers of Th17 cells were significantly increased in MPE, and that the accumulation of Th17 cells in MPE predicted improved patient survival [11]. It has been reported that human Tregs can differentiate into Th17 cells, when stimulated by allogeneic antigen-presenting cells in the presence of IL-2 or/and IL-15 [12].

It has been well documented that TGF-β is synthesized in cells as a pro-TGF-β precursor. Following homodimerization, pro-TGF-β is cleaved into two fragments: the C-terminal homodimer corresponds to mature TGF-β, while the N-terminal homodimer is latency-associated peptide (LAP) [13]. Mature TGF-β and LAP remain non-covalently bound to each other in a complex called latent TGF-β. Latent TGF-β is inactive because LAP prevents mature TGF-β from binding to its receptor, and hence from transducing a signal [14]. The role of TGF-β in the differentiation of human Th17 cells is still controversial. Some studies demonstrated that TGF-β is required for human Th17 cell differentiation [15,16], however, the other data showed that TGF-β suppresses the differentiation of Th17 cells [17,18]. Because TGF-β induces FOXP3 expression in naïve CD4^+ ^T cells and converts them to Tregs [19], while Tregs express cell surface or secrete TGF-β [20,21], this introduces the possibility that Tregs may be playing a role in generation and differentiation of Th17 cells via a LAP-dependent mechanism. In the present study, we were prompted to investigate whether CD39^+^Tregs are capable of suppressing generation and differentiation of Th17 cells, as well as whether LAP is involved in such a possible suppression in MPE.

## Methods

### Subjects

The study protocol was approved by our institutional review board for human studies, and informed consent was obtained from all subjects. Pleural fluid samples were collected from 16 patients (age range: 31 to 76 yr) with newly diagnosed lung cancer with MPE. Histologically, 11 cases were adenocarcinoma and 5 were squamous cell carcinoma. A diagnosis of MPE was established by demonstration of malignant cells in pleural fluid or/and on closed pleural biopsy specimen. The patients were excluded if they had received any invasive procedures directed into the pleural cavity or if they had suffered chest trauma within 3 mo prior to hospitalization. At the time of sample collection, none of the patients had received any anti-cancer therapy, corticosteroids, or other nonsteroid anti-inflammatory drugs.

### Sample Collection and Processing

The pleural fluid samples were collected in heparin-treated tubes from each subject, using a standard thoracocentesis technique within 24 h after hospitalization. Twenty milliliters of peripheral blood were drawn simultaneously. MPE specimens were immersed in ice immediately and were then centrifuged at 1,200 g for 5 min. The cell-free supernatants of MPE and serum were frozen at -80°C immediately after centrifuge for later determining cytokine concentrations. The cell pellets of MPE were resuspended in HBSS, and mononuclear cells were isolated by Ficoll-Hypaque gradient centrifugation (Pharmacia, Uppsala, Sweden) to determine the T cell subsets within 1 h. A pleural biopsy was performed when the results of pleural fluid analysis were suggestive of malignancy.

### Flow Cytometry

The expression markers on T cells from MPE and blood were determined by flow cytometry after surface staining or intracellular staining with anti-human-specific Abs conjugated with either phycoerythrin or fluorescein isothiocyanate. These human Abs included anti-CD3, anti-CD4, anti-CD39, anti-CD45RA, anti-CD45RO, anti-CD127, anti-LAP, anti-IL-17, and anti-FOXP3 mAbs, which were purchased from BD Biosciences or eBioscience (San Diego, CA). Intracellular staining for IL-17 or FOXP3 was performed on T cells stimulated with phorbol myristate acetate (50 ng/ml; Sigma-Aldrich) and ionomycin (1 μM; Sigma-Aldrich) in the presence of GolgiStop (BD Biosciences) for 5 h, and the intracellular IL-17 or FOXP3 was then stained with anti-IL-17 or -FOXP3 conjugated with phycoerythrin (eBioscience). Flow cytometry was performed on a BD FACSCalibur flowcytometer using FCS ExpressV3 software.

### Cell Isolation

Bulk CD4^+ ^T cells from pleural fluid and blood were isolated by negative selection (by depletion of CD8^+^, CD11b^+^, CD16^+^, CD19^+^, CD36^+^, and CD56^+ ^cells) with the Untouched CD4^+ ^cell isolation kit (Miltenyi Biotec, Auburn, CA) according to the manufacturer's instructions. After isolation of bulk CD4^+ ^T cells, the naïve CD4^+ ^T cells (CD45RA^+^CD45RO^-^) were further purified by EasySep enrichment kits (StemCell Technologies, Vancouver, British Columbia, Canada) according to the manufacturer's instructions. The purity of naïve CD4^+ ^T cells was > 97%, as measured by flow cytometry.

CD4^+ ^T cells were also stained with CD4-PerCP-Cy5.5, CD25-PE, and CD39-FITC, (eBiosciences), and CD4^+^CD25^high^CD39^+ ^T cells and CD4^+^CD25^- ^responder T cells were sorted using a Beckman Coulter cell sorter. Purity of the sorted populations was > 97%.

### Generation and Differentiation of Th17 Cells and Tregs in MPE

Purified naïve CD4^+ ^T cells (5 × 10^5^) were cultured in 1 ml of complete medium containing human IL-2 (2 ng/ml) in 48-well plates and stimulated with plate-bound anti-CD3 (OKT3; 1 μg/ml) and soluble anti-CD28 mAbs (1 μg/ml) for 7 d. The exogenous cytokines used were TGF-β1 (5 ng/ml), IL-1β (10 ng/ml), IL-6 (100 ng/ml), and IL-23 (10 ng/ml). Recombinant human IL-1β, IL-2, IL-6, IL-23, and TGF-β1, were purchased from R&D Systems. In some experiments, designated numbers of CD39^+^Tregs were added into the cultures. To demonstrate that LAP was responsible for the inhibitive effects of CD39^+^Tregs, blocking experiments were performed by mixing the MPE with 500 ng/ml of anti-LAP mAb (Clone 27235) or mouse IgG irrelevant isotype control (R&D Systems). The culture supernatants were collected for determining IL-17 concentration.

### Measurement of Cytokines

The concentrations of IL-1β, IL-6, IL-23, and TGF-β1 in both pleural fluids and sera, as well as IL-17 in culture supernatants, were measured by sandwich ELISA kits according to the manufacturer's protocols (all kits were purchased from R & D Systems Inc., Minneapolis, MN, USA). All samples were assayed in duplicate. The lower detection limits of IL-1β, IL-6, IL-23, TGF-β1, and IL-17 were 1 pg/ml, 0.70 pg/ml, 6.8 pg/ml, 4.61 pg/ml, and 15 pg/ml, respectively.

### Statistics

Data are expressed as mean ± SEM. Comparisons of the data between different groups were performed using a Kruskal-Wallis one-way analysis of variance on ranks. For data in MPE and in the corresponding blood, paired data comparisons were made using a Wilcoxon signed-rank test. Analysis was completed with SPSS version 16.0 Statistical Software (Chicago, IL, USA), and p values of less than 0.05 were considered to indicate statistical significance.

## Results

### Tregs and Th17 Cells Were Significantly Increased in MPE

**Figure 1:**
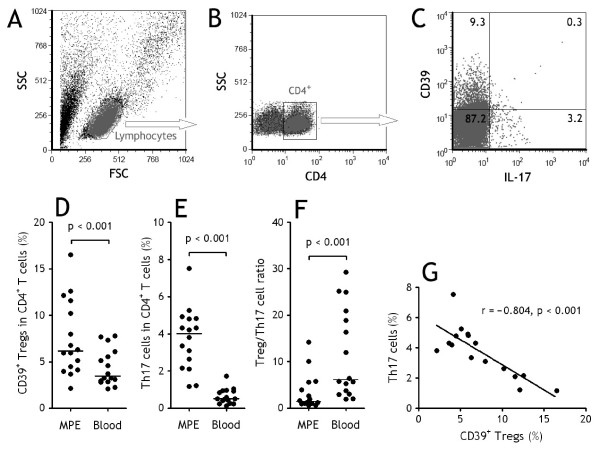
**Both CD39^+ ^regulatory T (Tregs) cells and Th17 cells increased in malignant pleural effusion (MPE)**. (A) Lymphocytes were identified based on their characteristic properties shown in the forward scatter (FSC) and sideward scatter (SSC). (B) A representative gating was set for CD4^+ ^T cells from pleural lymphocytes. (C) A representative dot plots showing expression of CD39 and IL-17 in pleural CD4^+ ^T cells. Comparisons of percentages of CD39^+^Tregs (D), Th17 cells (E), and ratios of CD39^+^Tregs/Th17 cells (F) in MPE and blood from patients with lung cancer (n = 16). The percentages of CD39^+^Tregs and Th17 cells were determined by flow cytometry. Horizontal bars indicate medians. Comparison was made using a Wilcoxon signed-rank test. (G) The percentages of Th17 cells correlated with CD39^+^Tregs cells in MPE. Correlations were determined by Spearman's rank correlation coefficients.

We first used flow cytometry to identify both CD39^+ ^Tregs and Th17 cells in CD4^+ ^T cells in MPE (Figure 1A, B, C) and peripheral blood. With gating on CD4^+^CD25^high ^subset, we once again observed in the present study that a significant increase in CD39^+^Tregs were was observed in MPE (7.5 ± 1.0%) compared with blood (4.4 ± 0.5%) (n = 16, Wilcoxon signed-rank test, p < 0.001) (Figure 1D). Consistent with our previous findings [14], we noted that percentages of Th17 cells represented the higher values in MPE (3.7 ± 0.4%), showing a significant increase in comparison with those in the corresponding blood (0.6 ± 0.1%) (n = 16, Wilcoxon signed-rank test, p < 0.001) (Figure 1E). We further found that the ratios of CD39^+^Tregs/Th17 cells were significantly lower in MPE (3.3 ± 1.0) than in blood (11.5 ± 2.4, n = 16, Wilcoxon signed-rank test, p < 0.001) (Figure 1F). In addition, pleural Th17 cell numbers were correlated negatively with Treg numbers (r = -0.804, p < 0.001) (Figure 1G).

**Figure 2:**
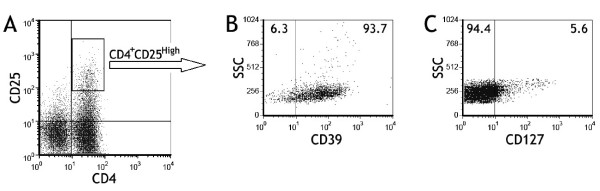
**CD39 and CD127 expressed on CD4^+^CD25^high ^T cells**. The subset of pleural CD4^+^CD25^high ^T cells (A) was identified by flow cytometry for determining the surface expression of CD39 (B) and CD127 (C), data for one representative donor of 16 tested are shown.

As show in Figure 2, majority of CD4^+^CD25^high ^T cells were CD39 positive (86 - 94%) and were CD127 negative (88 - 95%), possessing typical phenotypes of Tregs.

### Impacts of Cytokines on Tregs and Th17 Cells in MPE

**Figure 3:**
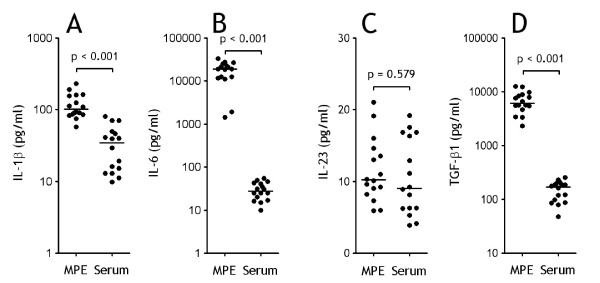
**Proinflammatory cytokines increased in malignant pleural effusion (MPE)**. Comparisons of concentrations of IL-1β (A), IL-6 (B), IL-23 (C), and TGF-β1 (D) in both MPE and sera from patients with lung cancer (n = 16). The cytokines were determined by ELISA, and comparisons of cytokine concentrations were made using a Wilcoxon signed-rank test.

We determined some cytokines that reported be involved in generation and differentiation of Tregs or Th17 cells, and observed that high levels of IL-1β, IL-6, and TGF-β1, but not of IL-23, in MPE when compared with the corresponding sera (Figure 3), suggesting that these proinflammatory cytokines might affect the generation and differentiation of Tregs or/and Th17 cells in MPE.

**Figure 4:**
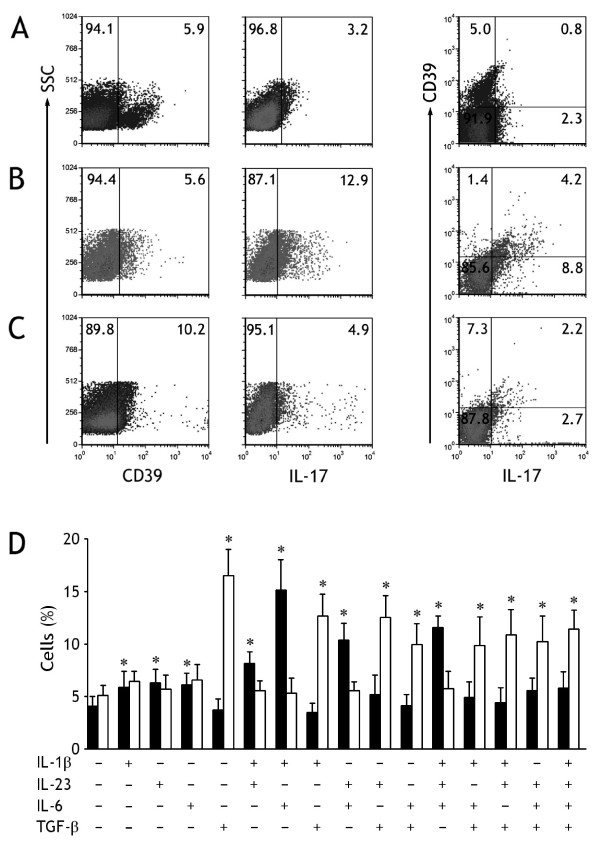
**Generation and differentiation of human CD39^+^Tregs and Th17 cells from malignant pleural effusion regulated by different cytokines**. (A) The representative dot plots of freshly isolated naïve CD4^+ ^T cells from malignant pleural effusion were determined for expression of CD39 and IL-17 by flow cytometry. (B) The representative dot plots of CD39^+^Tregs and Th17 cells detected in naïve CD4^+ ^T cells after culturing in presence both IL-1β and IL-6. (C) The representative dot plots of CD39^+^Tregs and Th17 cells detected in naïve CD4^+ ^T cells after culturing in presence of TGF-β1. (D) The mean ± SEM of CD39^+^Tregs (open bars) and Th17 cells (closed bars) detected in naïve CD4^+ ^T cells from 5 independent experiments. The purified naïve CD4^+ ^T cells were stimulated with plate-bound anti-CD3 and soluble anti-CD28 mAbs in the presence of the indicated cytokines, either alone or in various combinations for 7 d. * p < 0.01 compared with their corresponding controls with no cytokines.

To evaluate the contribution of cytokines to the numbers of pleural CD39^+^Tregs and Th17 cells, we purified naïve CD4^+ ^T cells from MPE and blood and cultured them in the presence of one or more of IL-1β, IL-6, IL-23, and TGF-β. With IL-2-containing medium provided a baseline for comparison, IL-1β, IL-6, or IL-23, but not TGF-β, could promote the differentiation of Th17 cells from naïve CD4^+ ^T cells (Figure 4). The combination of IL-1β plus IL-6, IL-1β plus IL-23, IL-6 plus IL-23, or IL-1β plus IL-6 plus IL-23, significantly increased the percentage of Th17 cells at higher extents compared with any single one of above cytokines. Although a significant high concentration of TGF-β was found in MPE, it did not promote the generation and differentiation and of Th17 cells; in contrast, TGF-β could reduce the increased percentage of Th17 cells stimulated by the above cytokines. On the other hand, TGF-β was capable of promoting the differentiation of CD39^+^Tregs during the 7-day culture, and that any one or their various combinations of IL-1β, IL-6, and IL-23 did not affect the increase in CD39^+^Treg numbers induced by TGF-β (Figure 4).

### Inhibition of Generation and Differentiation of Th17 Cells by CD39^+^Tregs

**Figure 5:**
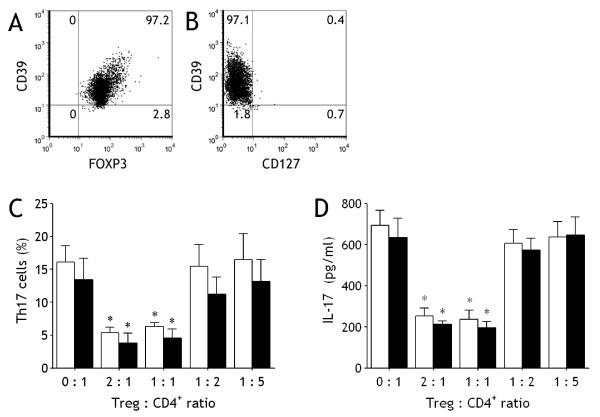
**CD39^+^Tregs inhibit generation and differentiation of Th17 cells**. The representative dot plots showing isolated pleural CD39^+^CD4^+^CD25^high ^T cells are almost CD39 positive (A) and CD127 negative (B). Naïve CD4^+ ^T cells isolated from malignant pleural effusion (open bars) and blood (closed bars) were cultured in the conditions described in Figure 4 with indicated ratio of CD39^+^Tregs, Th17 cell numbers were determined by flow cytometry (C) and IL-17 concentrations in the cultured supernatants were determined by ELISA (D). The results are reported as mean ± SEM from 5 independent experiments. * p < 0.01 compared with naïve CD4^+ ^T cells without CD39^+^Tregs.

On the basis of our observation that both CD39^+^Tregs and Th17 cells were significantly increased in MPE, and that pleural CD39^+^Treg numbers were correlated negatively with pleural Th17 cell numbers, we therefore investigated the impacts of Tregs on generation and differentiation of Th17 cells *in vitro*. We purified CD39^+^CD4^+^CD25^high ^T cells from both MPE and blood, and found that this subset of T cells were almost FOXP3 positive (> 97%) (Figure 5A) and were almost CD127 negative (> 97%) (Figure 5B), possessing typical phenotypes of Tregs. As shown in Figure 5C, generation and differentiation of Th17 cells were observed when the purified naïve CD4^+ ^T cells were cultured for 7 d in presence of IL-1β and IL-6. When CD39^+^Tregs were added into the coculture, Th17 cell numbers decreased as CD39^+^Treg numbers increased. Likewise, IL-17 concentrations in the cultured supernatants decreased as CD39^+^Treg numbers increased (Figure 5D). There were no differences in inhibiting effects on both Th17 cell numbers and IL-17 concentrations between pleural CD39^+^Tregs and blood CD39^+^Tregs (Figure 5C and 5D).

### LAP Mediates Treg-Induced Inhibition of Th17 Cells

**Figure 6:**
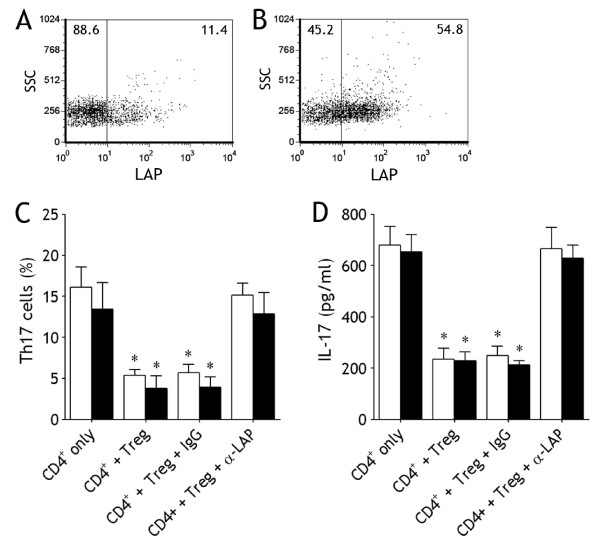
**LAP mediates Treg-induced inhibition of Th17 cells**. Freshly purified pleural CD39^+^Tregs (A) and cultured CD39^+^Tregs (B) were analyzed by flow cytometry for determining the surface expression of LAP, data for one representative donor of 5 tested are shown. Naïve CD4^+ ^T cells isolated from malignant pleural effusion (open bars) and blood (closed bars) were cultured CD39^+^Tregs (ratio, 1 : 1), an anti-LAP mAb or isotype control IgG was added into the coculture, Th17 cell numbers were determined by flow cytometry (C) and IL-17 concentrations in the cultured supernatants were determined by ELISA (D). The results are reported as mean ± SEM from 5 independent experiments. * p < 0.01 compared with isotype control.

Since high concentration of TGF-β1 was found in MPE (Figure 2D), we explored whether LAP was involved in the observed suppressive effect by CD39^+^Tregs on the generation and differentiation of Th17 cells. We determined LAP expression of on the cell surface of CD39^+^Tregs by flow cytometry and found that there was some an extent of LAP surface expression on fleshly purified CD39^+^Tregs (6.2 - 12.4%) (Figure 6A); when CD39^+^Tregs were cultured with plate-bound anti-CD3 and soluble anti-CD28 mAbs in the presence of IL-1β and IL-6 for 7 d, the expression of LAP increased significantly (47.2 - 56.3%) (Figure 6B). We included a blocking mAb against LAP in the above coculture of naïve CD4^+ ^T cells and CD39^+^Tregs from MPE or blood. As shown in Figure 6C, addition of the anti-LAP mAb to the cultures markedly reverted the inhibitory effect exerted by CD39^+^Tregs. Therefore, the above results indicate that CD39^+^Tregs inhibit generation and differentiation of Th17 cells via a LAP-dependent mechanism. In addition, a similar inhibitory effect of CD39^+^Tregs on IL-17 production was also observed (Figure 6D).

## Discussion

In the previous studies, we have reported that increased Tregs and Th17 cells could be found in MPE [9-11]. In the present study, we have extended the previous works and demonstrated that CD39^+^Tregs play an important role in regulating generation and differentiation of Th17 cells in human MPE.

It has been reported by the other groups [15,22-24] that Tregs can differentiate into Th17 cells. In the animal studies, the development and differentiation of Th17 cells was described to be linked to that of Tregs in a reciprocal fashion, both TGF-β and IL-6 appeared obligatory for this differentiation process [25,26]. Murine activated Tregs promoted Th17 cell differentiation from CD4^+ ^T cells likely through their production of TGF-β [26,27]. However, the process of human Tregs differentiating into Th17 cells was enhanced by exogenous cytokines, such as IL-1β, IL-6, IL-21, and IL-23, and inhibited by TGF-β [15,22]. The IL-17-producing Tregs strongly inhibit the proliferation of CD4^+ ^responder T cells, and maintain their suppressive function via a cell-cell contact mechanism [23,24]. These data suggest that in addition to their well-known suppressive functions, these Tregs likely play additional, as yet undescribed, proinflammatory functions. The ability of Tregs to secrete IL-17 may represent inherent plasticity in this population to convert to effector T cells under conditions of inflammation, such as in the presence of IL-2 or IL-15 (14), IL-1β and IL-6 [22], IL-1β and IL-2 (28), or dendritic cells activated under specific conditions [29,30].

Consistent with the findings reported by other authors [31,32], we also found fewer Th17 cells than CD39^+^Tregs in MPE, although the numbers of both CD39^+^Tregs and Th17 cells were increased in MPE when compared with peripheral blood. Interestingly, we further noted that the numbers of CD39^+^Tregs and Th17 cells are inversely correlated in MPE, and that the ratios of CD39^+^Tregs/Th17 cells were significantly lower in MPE than in blood, suggesting that there could be a dynamic interaction between Th17 cells and CD39^+^Tregs in the tumor microenvironment. Therefore, we were prompted to investigate whether CD39^+^Tregs are capable of suppressing generation and differentiation of Th17 cells. It was quite well documented that various cytokines contribute to the generation and differentiation of Tregs or Th17 cells. In the present study, high levels of IL-1β, IL-6, TGF-β1, but not of IL-23, could be found in MPE, moreover, CD39^+^Tregs could express LAP on their cell surface. Our results suggested that these proinflammatory cytokines, especially TGF-β, might affect the generation and differentiation of Tregs or/and Th17 cells in MPE. Indeed, we found that IL-1β, IL-6, or IL-23, but not TGF-β, could promote the differentiation of Th17 cells from naïve CD4^+ ^T cells, and the combination of IL-1β plus IL-6, IL-1β plus IL-23, IL-6 plus IL-23, or IL-1β plus IL-6 plus IL-23, significantly increased the percentage of Th17 cells at higher extents compared with any single one of above cytokines. On the other hand, TGF-β could reduce the increased percentage of Th17 cells stimulated by the above cytokines. In contrast, TGF-β could promote the differentiation of CD39^+^Tregs under the same conditions.

Tregs in human studies have been being identified mostly based on high expression of CD25 and FOXP3 and, in some cases, low expression of CD127 [9,10,33]. However, FOXP3 mRNA expression could be induced in human CD25^- ^and CD8^+ ^peripheral blood mononuclear cells, which were both negative for FOXP3 mRNA expression after isolation, indicating that FOXP3 expression in humans, unlike mice, may not be specific for Tregs and may be only a consequence of activation status [34]. Furthermore, these markers cannot be used to identify Treg poststimulation *in vitro*, since their expression patterns change toward the Treg phenotype upon activation of effector T cells. Recently, CD39 was found to be expressed on a subpopulation of Tregs [35,36]. The technique of isolating human Tregs based on the CD39 expression has been proved to be highly desirable [37]. The advantage of this marker is that it recognizes Tregs with suppressor activity mediated via pericellular adenosine, which is the end product of enzymatic degradation of ATP [38]. Thus, CD39 defines Treg based not only on the phenotypic but also functional characteristics. In the present study, we isolated Tregs from MPE and blood based CD39 expression and found that the purified CD39^+^CD4^+^CD25^high ^T cells were almost FOXP3 positive and were almost CD127 negative, indicating that these T cells were Tregs. The most important finding in the present study was that CD39^+^Tregs could be able to inhibit the generation and differentiation of Th17 cells in a dose-dependent manner.

The mechanism by which human Tregs inhibit the generation and differentiation of Th17 cells is unknown. It was reported that murine Tregs inhibit Th17 cell responses *in vivo *in a signal transducer and activator of transcription-3-dependent manner, and Treg cell-specific ablation of signal transducer and activator of transcription-3 leads to the loss of their suppressive functions [39]. Fletcher et al [40] have demonstrated for the first time that human Tregs can suppress IL-17 production by responder T cells, their data suggested that CD39 molecule might be involved in the mechanism by which Tregs suppress generation and differentiation of Th17, since the hydrolysis of ATP by CD39 could reduce IL-17 production by CD4^+ ^T cells, and an analog of adenosine, the final breakdown product of ATP effectively inhibited IL-17. As above mentioned, high concentration of TGF-β was found in MPE, and majority of pleural CD39^+^Tregs expressed LAP on their surface, we thus explored whether LAP was involved in the observed suppressive effect by CD39^+^Tregs on the generation and differentiation of Th17 cells. In the *in vitro *coculture of naïve CD4^+ ^T cells and CD39^+^Tregs, We added a blocking mAb against LAP and observed that this mAb was able to revert the inhibitory effect exerted by CD39^+^Tregs. Thus, we herein provided the direct evidence for the first time that CD39^+^Tregs inhibit generation and differentiation of Th17 cells via a LAP-dependent mechanism.

In conclusion, our data showed that both CD39^+^Tregs and Th17 cells were increased in MPE when compared with blood, the numbers of CD39^+^Tregs were correlated negatively with those of Th17 cells, and that CD39^+^Tregs inhibit generation and differentiation of pleural Th17 cells via a LAP-dependent mechanism.

## Conclusions

This study showed that both CD39^+^Tregs and Th17 cells were increased in MPE when compared with blood, the numbers of CD39^+^Tregs were correlated negatively with those of Th17 cells, and that CD39^+^Tregs inhibit generation and differentiation of pleural Th17 cells via a LAP-dependent mechanism.

## Competing interests

The authors declare that they have no competing interests.

## Authors' contributions

YZJ, QZ, and HZS designed the study design and the experiments. JCZ and XL were responsible for flow cytometry and data collection. CW and SMQ analyzed the data. JBX and HZS drafted the manuscript. YZJ, QZ, JCZ and XL read, critically revised and all authors approved the final manuscript.

## Funding

This study was supported by a grant from National Science Fund for Distinguished Young Scholars (No. 30925032) and by grants from National Natural Science Foundation of China (No. 30872343).

## References

[B1] Light RW Clinical practice. Pleural effusion N Engl J Med 2002 346 1971 1977 10.1056/NEJMcp010731 12075059

[B2] Lucivero G Pierucci G Bonomo L Lymphocyte subsets in peripheral blood and pleural fluid Eur Respir J 1988 1 337 340 3260873

[B3] Ikeda H Chamoto K Tsuji T Suzuki Y Wakita D Takeshima T Nishimura T The critical role of type-1 innate and acquired immunity in tumor immunotherapy Cancer Sci 2004 95 697 703 10.1111/j.1349-7006.2004.tb03248.x 15471553 PMC11159994

[B4] Atanackovic D Block A de WA Faltz C Hossfeld DK Hegewisch-Becker S Characterization of effusion-infiltrating T cells: benign versus malignant effusions Clin Cancer Res 2004 10 2600 2608 10.1158/1078-0432.CCR-03-0239 15102661

[B5] Hori S Nomura T Sakaguchi S Control of regulatory T cell development by the transcription factor Foxp3 Science 2003 299 1057 1061 10.1126/science.1079490 28115586

[B6] Fontenot JD Gavin MA Rudensky AY Foxp3 programs the development and function of CD4^+^CD25^+ ^regulatory T cells Nat Immunol 2003 4 330 336 12612578 10.1038/ni904

[B7] Mills KH Induction, function and regulation of IL-17-producing T cells Eur J Immunol 2008 38 2636 2649 10.1002/eji.200838535 18958872

[B8] Weaver CT Hatton RD Interplay between the T_H_17 and T_Reg _cell lineages: a (co-)evolutionary perspective Nat Rev Immunol 2009 9 883 889 10.1038/nri2660 19935807

[B9] Chen YQ Shi HZ Qin XJ Mo WN Liang XD Huang ZX Yang HB Wu C CD4^+^CD25^+ ^regulatory T lymphocytes in malignant pleural effusion Am J Respir Crit Care Med 2005 172 1434 1439 10.1164/rccm.200504-588OC 16151041

[B10] Qin XJ Shi HZ Liang QL Liu GN Jiang J Qin SM Deng JM Ye ZJ CCL22 recruits CD4-positive CD25-positive regulatory T cells into malignant pleural effusion Clin Cancer Res 2009 15 2231 2237 10.1158/1078-0432.CCR-08-2641 19318474

[B11] Ye ZJ Zhou Q Gu YY Qin SM Ma WL Xin JB Tao XN Shi HZ Generation and differentiation of interleukin-17-producing CD4^+ ^T cells in malignant pleural effusion J Immunol 2010 185 6348 6354 10.4049/jimmunol.1001728 20952674

[B12] Koenen HJ Smeets RL Vink PM van Rijssen E Boots AM Joosten I Human CD25^high^Foxp3^pos ^regulatory T cells differentiate into IL-17-producing cells Blood 2008 112 2340 2352 10.1182/blood-2008-01-133967 18617638

[B13] Gleizes PE Munger JS Nunes I Harpel JG Mazzieri R Noguera I Rifkin DB TGF-beta latency: biological significance and mechanisms of activation Stem Cells 1997 15 190 197 10.1002/stem.150190 9170210

[B14] Lawrence DA Latent-TGF-beta: an overview Mol Cell Biochem 2001 219 163 170 10.1023/A:1010819716023 11354248

[B15] Volpe E Servant N Zollinger R Bogiatzi SI Hupé P Barillot E Soumelis V A critical function for transforming growth factor-β, interleukin 23 and proinflammatory cytokines in driving and modulating human T_H_-17 responses Nat Immunol 2008 9 650 657 18454150 10.1038/ni.1613

[B16] Yang L Anderson DE Baecher-Allan C Hastings WD Bettelli E Oukka M Kuchroo VK Hafler DA IL-21 and TGF-β are required for differentiation of human T_H_17 cells Nature 2008 454 350 352 10.1038/nature07021 18469800 PMC2760130

[B17] Acosta-Rodriguez EV Napolitani G Lanzavecchia A Sallusto F Interleukins 1β and 6 but not transforming growth factor-β are essential for the differentiation of interleukin 17-producing human T helper cells Nat Immunol 2007 8 942 949 17676045 10.1038/ni1496

[B18] Miyahara Y Odunsi K Chen W Peng G Matsuzaki J Wang RF Generation and regulation of human CD4^+ ^IL-17-producing T cells in ovarian cancer Proc Natl Acad Sci USA 2008 105 15505 15510 10.1073/pnas.0710686105 18832156 PMC2563129

[B19] Chen W Konkel JE TGF-β and 'adaptive' Foxp3^+ ^regulatory T cells J Mol Cell Biol 2010 2 30 36 10.1093/jmcb/mjp004 19648226 PMC2905060

[B20] Nakamura K Kitani A Strober W Cell contact-dependent immunosuppression by CD4^+^CD25^+ ^regulatory T cells is mediated by cell surface-bound transforming growth factor beta J Exp Med 2001 194 629 644 10.1084/jem.194.5.629 11535631 PMC2195935

[B21] Oida T Xu L Weiner HL Kitani A Strober W TGF-β-mediated suppression by CD4^+^CD25^+ ^T cells is facilitated by CTLA-4 signaling J Immunol 2006 177 2331 2339 16887994 10.4049/jimmunol.177.4.2331

[B22] Beriou G Costantino CM Ashley CW Yang L Kuchroo VK Baecher-Allan C Hafler DA IL-17-producing human peripheral regulatory T cells retain suppressive function Blood 2009 113 4240 4249 10.1182/blood-2008-10-183251 19171879 PMC2676084

[B23] Voo KS Wang YH Santori FR Boggiano C Wang YH Arima K Bover L Hanabuchi S Khalili J Marinova E Zheng B Littman DR Liu YJ Identification of IL-17-producing FOXP3^+ ^regulatory T cells in humans Proc Natl Acad Sci USA 2009 106 4793 4798 10.1073/pnas.0900408106 19273860 PMC2653560

[B24] Ayyoub M Deknuydt F Raimbaud I Dousset C Leveque L Bioley G Valmori D Human memory FOXP3^+ ^Tregs secrete IL-17 ex vivo and constitutively express the T_H_17 lineage-specific transcription factor RORγt Proc Natl Acad Sci USA 2009 106 8635 8640 10.1073/pnas.0900621106 19439651 PMC2688993

[B25] Bettelli E Carrier Y Gao W Korn T Strom TB Oukka M Weiner HL Kuchroo VK Reciprocal developmental pathways for the generation of pathogenic effector T_H_17 and regulatory T cells Nature 2006 441 235 238 10.1038/nature04753 16648838

[B26] Veldhoen M Hocking RJ Atkins CJ Locksley RM Stockinger B TGF-β in the context of an inflammatory cytokine milieu supports de novo differentiation of IL-17-producing T cells Immunity 2006 24 179 189 10.1016/j.immuni.2006.01.001 16473830

[B27] Xu L Kitani A Fuss I Strober W Cutting edge: regulatory T cells induce CD4^+^CD25^+^Foxp3^+ ^T cells or are self-induced to become Th17 cells in the absence of exogenous TGF-β J Immunol 2007 178 6725 6729 17513718 10.4049/jimmunol.178.11.6725

[B28] Deknuydt F Bioley G Valmori D Ayyoub M IL-1β and IL-2 convert human Treg into T_H_17 cells Clin Immunol 2009 131 298 307 10.1016/j.clim.2008.12.008 19211307

[B29] Osorio F LeibundGut-Landmann S Lochner M Lahl K Sparwasser T Eberl G Reis e Sousa C DC activated via dectin-1 convert Treg into IL-17 producers Eur J Immunol 2008 38 3274 3281 10.1002/eji.200838950 19039774 PMC2699423

[B30] Radhakrishnan S Cabrera R Schenk EL Nava-Parada P Bell MP Van Keulen VP Marler RJ Felts SJ Pease LR Reprogrammed FoxP3^+ ^T regulatory cells become IL-17^+ ^antigen-specific autoimmune effectors in vitro and in vivo J Immunol 2008 181 3137 3147 18713984 10.4049/jimmunol.181.5.3137 PMC2582200

[B31] Curiel TJ Coukos G Zou L Alvarez X Cheng P Mottram P Evdemon-Hogan M Conejo-Garcia JR Zhang L Burow M Zhu Y Wei S Kryczek I Daniel B Gordon A Myers L Lackner A Disis ML Knutson KL Chen L Zou W Specific recruitment of regulatory T cells in ovarian carcinoma fosters immune privilege and predicts reduced survival Nat Med 2004 10 942 949 10.1038/nm1093 15322536

[B32] Kryczek I Banerjee M Cheng P Vatan L Szeliga W Wei S Huang E Finlayson E Simeone D Welling TH Chang A Coukos G Liu R Zou W Phenotype, distribution, generation, and functional and clinical relevance of Th17 cells in the human tumor environments Blood 2009 114 1141 1149 10.1182/blood-2009-03-208249 19470694 PMC2723011

[B33] Liu W Putnam AL Xu-Yu Z Szot GL Lee MR Zhu S Gottlieb PA Kapranov P Gingeras TR Fazekas de St Groth B Clayberger C Soper DM Ziegler SF Bluestone JA CD127 expression inversely correlates with FoxP3 and suppressive function of human CD4^+ ^T reg cells J Exp Med 2006 203 1701 1711 10.1084/jem.20060772 16818678 PMC2118339

[B34] Morgan ME van Bilsen JH Bakker AM Heemskerk B Schilham MW Hartgers FC Elferink BG van der Zanden L de Vries RR Huizinga TW Ottenhoff TH Toes RE Expression of FOXP3 mRNA is not confined to CD4^+^CD25^+ ^T regulatory cells in humans Hum Immunol 2005 66 13 20 15620457 10.1016/j.humimm.2004.05.016

[B35] Borsellino G Kleinewietfeld M Di Mitri D Sternjak A Diamantini A Giometto R Höpner S Centonze D Bernardi G Dell'Acqua ML Rossini PM Battistini L Rötzschke O Falk K Expression of ectonucleotidase CD39 by Foxp3^+ ^Treg cells: hydrolysis of extracellular ATP and immune suppression Blood 2007 110 1225 1232 10.1182/blood-2006-12-064527 17449799

[B36] Deaglio S Dwyer KM Gao W Friedman D Usheva A Erat A Chen JF Enjyoji K Linden J Oukka M Kuchroo VK Strom TB Robson SC Adenosine generation catalyzed by CD39 and CD73 expressed on regulatory T cells mediates immune suppression J Exp Med 2007 204 1257 1265 10.1084/jem.20062512 17502665 PMC2118603

[B37] Mandapathil M Lang S Gorelik E Whiteside TL Isolation of functional human regulatory T cells (Treg) from the peripheral blood based on the CD39 expression J Immunol Methods 2009 346 55 63 10.1016/j.jim.2009.05.004 19450601 PMC2703678

[B38] Robson SC Sévigny J Zimmermann H The E-NTPDase family of ectonucleotidases: Structure function relationships and pathophysiological significance Purinergic Signal 2006 2 409 430 10.1007/s11302-006-9003-5 18404480 PMC2254478

[B39] Chaudhry A Rudra D Treuting P Samstein RM Liang Y Kas A Rudensky AY CD4^+ ^regulatory T cells control T_H_17 responses in a Stat3-dependent manner Science 2009 326 986 991 10.1126/science.1172702 19797626 PMC4408196

[B40] Fletcher JM Lonergan R Costelloe L Kinsella K Moran B O'Farrelly C Tubridy N Mills KH CD39^+^Foxp3^+ ^regulatory T Cells suppress pathogenic Th17 cells and are impaired in multiple sclerosis J Immunol 2009 183 7602 7610 10.4049/jimmunol.0901881 19917691

